# 2-Substituted-3-(5-Substituted-1,3,4-oxadiazol/thiadiazol-2-yl) Thiazolidin-4-one Derivatives: Synthesis, Anticancer, Antimicrobial, and Antioxidant Potential

**DOI:** 10.3390/ph16060805

**Published:** 2023-05-29

**Authors:** Davinder Kumar, Navidha Aggarwal, Harsh Kumar, Garima Kapoor, Aakash Deep, Shabana Bibi, Aastha Sharma, Hitesh Chopra, Rakesh Kumar Marwaha, Abdulrahman Alshammari, Metab Alharbi, Abdul Hayee

**Affiliations:** 1 Department of Pharmaceutical Sciences, Maharishi Dayanand University, Rohtak 124001, India; 2 MM College of Pharmacy, Maharishi Markandeshwar (Deemed to Be University), Mullana 133207, India; 3 KIET School of Pharmacy, KIET Group of institution Delhi-NCR, Ghaziabad 201206, India; 4 Department of Pharmaceutical Sciences, Chaudhary Bansi Lal University, Bhiwani 127021, India; 5 Department of Biosciences, Shifa Tameer-e-Millat University, Islamabad 41000, Pakistan; 6 Yunnan Herbal Laboratory, College of Ecology and Environmental Sciences, Yunnan University, Kunming 650091, China; 7 Chitkara College of Pharmacy, Chitkara University, Punjab 140401, India; 8 Department of Pharmacology and Toxicology, College of Pharmacy, King Saud University, Riyadh 11451, Saudi Arabia; 9 Department of Immunology, Faculty of Medicine, Academic Assembly, University of Toyama, Toyama 930-0194, Japan

**Keywords:** 1,3,4-oxadiazole, 1,3,4-thiadiazole, anticancer, synthesis, thiazolidin-4-one, antimicrobial activity, antioxidant potential

## Abstract

In this innovative research, a novel series of thiazolidin-4-one analogues having a 1,3,4-oxadiazole/thiadiazole moiety were derived and the structures of all the newly obtained molecules were established using different physicochemical and analytical means (^1^H-NMR, FTIR, mass spectra, and elemental analyses). The synthesized molecules were then investigated for their antiproliferative, antimicrobial, and antioxidant potential. The cytotoxicity screening studies revealed that analogues D-1, D-6, D-15, and D-16 possessed comparable efficacy, within the IC_50_ range (1 to 7 μM), when taking doxorubicin as a reference drug (IC_50_ = 0.5 μM). The antimicrobial activity was assessed using different Gram-(+) and Gram-(−) bacterial and fungal strains and the results revealed that molecules D-2, D-4, D-6, D-19, and D-20 possessed potent activity against selective strains of microbes with MIC ranges of 3.58 to 8.74 µM. The antioxidant evaluation was performed using the DPPH assay and the screening results revealed that analogue D-16 was the most potent derivative (IC_50_ = 22.3 µM) when compared with the positive control, ascorbic acid (IC_50_ = 111.6 µM). Structure–activity relationship (SAR) studies of the synthesized novel derivatives revealed that para-substituted halogen and hydroxy derivatives have remarkable potential against the MCF-7 cancer cell line and antioxidant potential. Similarly, electron-withdrawing groups (Cl/NO_2_) and -donating groups at the para position possess moderate to promising antimicrobial potential.

## 1. Introduction

Worldwide, cancer remains a significant health issue and is the most common cause of death. Uncontrolled abnormal cell proliferation can cause death if the ranges are not controlled. Throughout the world, cancer incidence and mortality rates are rising. Thus, this global burden of cancer, after cardiovascular diseases, is causing a significant decline in lifetimes and a rise in premature deaths [1,2]. Many different types of therapeutic approaches are being applied for the treatment of cancer. Several anticancer drugs are available around the world but most drugs are associated with large numbers of serious side effects, toxicity, and drug resistance [3,4]. Thus, it is crucial to develop novel anticancer molecules with great potential and fewer side effects.

**Figure 1:**
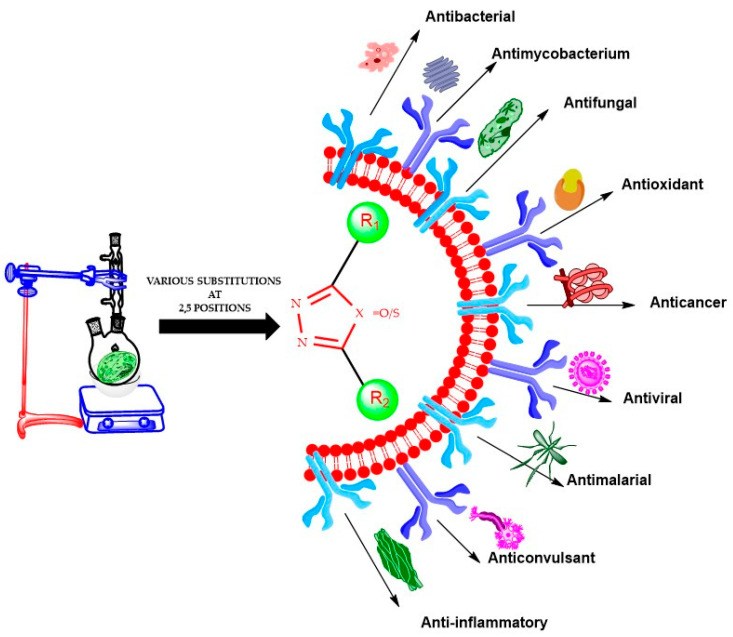
Biological potentials of 1,3,4-oxadiazoles/thiadiazoles.

Similarly, microbial infections also affect a large population around the world. The continuous use and overuse of clinical antimicrobial drugs has led to the emergence of microbial resistance, and pathogenic microorganisms have developed resistance toward most antimicrobial agents [4,5,6]. The prevalence of antibiotic resistance has increased and created a serious demand for new antimicrobial agents. It is necessary to develop new medicines to fight and prevent antibiotic resistance [7,8,9]. Similarly, the prevention of oxidative stress is crucial in reducing diseases, such as cancer and cardiovascular disease, but there is a need for more potent antioxidants with superior bioavailability. Many heterocyclic moieties are attracting great attention due to their fascinating antimicrobial, antioxidant, and anticancer activity. Among the heterocyclic moieties, nitrogen or sulfur atom-containing azole groups, e.g., 1,3,4-oxadiazoles/thiadiazoles, are of major interest for all researchers and scientists due to their potential biological activity as antimicrobial [10], antifungal [11,12], anticancer [13,14], antimalarial [15], antioxidant [16,17], anti-inflammatory [18], and anticonvulsant [19] agents, etc. (Figure 1).

**Figure 2:**
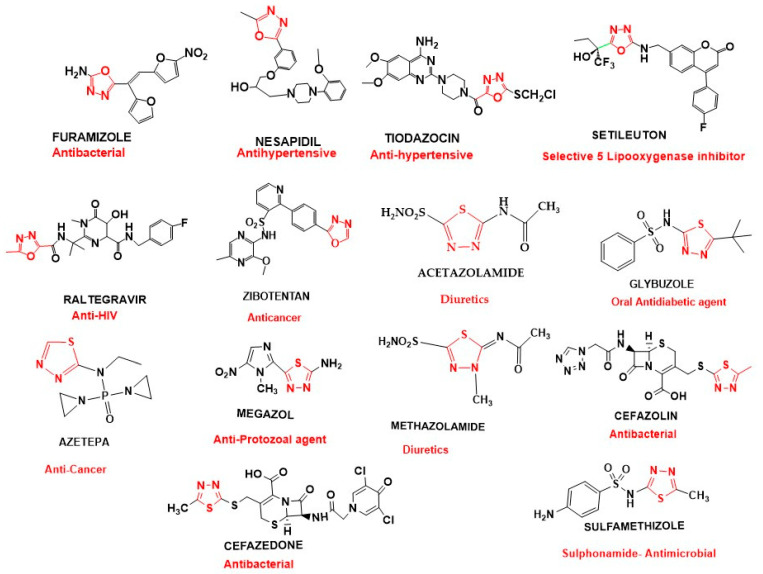
Various 1,3,4-oxadiazole/thiadiazole ring-containing drugs.

The 1,3,4-thiadiazole-containing drugs Cefazolin and Nefazodone (antibiotics, cell wall synthesis inhibitors), Megazol (antiprotozole, protein, and DNA synthesis inhibitor), Methazolamide and Acetazolamide (diuretics, carbonic anhydrase inhibitors) Sulphamethizole (antimicrobial, dihydropteroate synthase inhibitor), and Azetepa (anticancer, alkylating agent),and 1,3,4-oxadiazole-containing commercial drugs such as Furamizole (potent antibacterial action), Nesapidil (anti-arrhythmic action), Raltegravir (antiviral drug), Tiodazosin (antihypertensive agent), and the most promising FDA-approved derivative, the anticancer agent Zibotentan, are commercially available [20,21,22,23,24,25] (Figure 2).

From this perspective, 1,3,4-oxadiazole/thiadiazole conjugates in medicinal chemistry have been developed rapidly and represent a significant approach to address the various drawbacks associated with anticancer and antimicrobial drugs, such as drug toxicity, drug resistance, and other serious side effects [26,27,28]. The 1,3,4-thiadiazole and 1,3,4-oxadiazole conjugates have therefore been investigated extensively. Prompted by these findings, we explore the synthesis, anticancer efficacy, and antimicrobial and antioxidant potential of thiazolidin-4-one derivatives of 1,3,4-oxadiazole/thiadiazole rings.

## 2. Results 

**Scheme 1:**
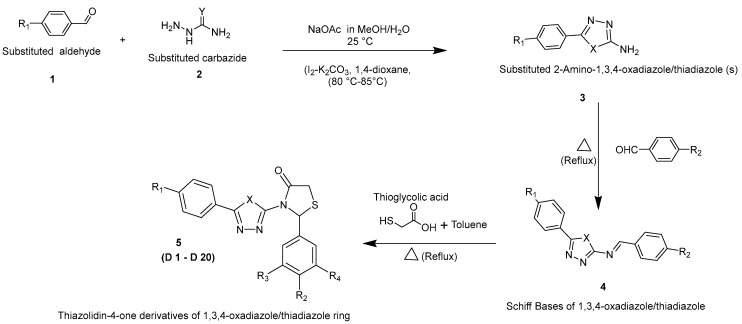
Synthesis of thiazolidin-4-one derivatives of 1,3,4-oxadiazole/thiadiazole ring.

All azole derivatives (1,3,4-oxadiazole/thiadiazoles) were synthesized using the synthetic method shown in Scheme 1 (Table 1). In the first step, a mixture containing semi/thiosemicarbazide (2) and sodium acetate was added to water and stirred well, followed by the addition of substituted aldehydes (1) in methanol at room temperature to provide an intermediate solid residue. The residue was redissolved in 1,4-dioxane and the stirring was continued (4–6 h) at 80–85 °C with the addition of K_2_CO_3_ and iodine in sequence to obtain 2-Amino 1,3,4-oxadiazole/thiadiazole derivatives (3). Schiff’s bases (5) were obtained by treating the substituted aldehydes (4) with 2-amino 1,3,4-oxadiazole/thiadiazole while taking acetic acid (glacial) as a catalyst. The final compounds (7) were obtained by reaction with thioglycolic acid (6) using a small quantity of zinc chloride as a catalyst. A variety of spectral techniques, i.e., FT-IR (KBr, cm^−1^), ^1^H-NMR (DMSO-*d*_6_, 400 MHz, δ ppm), mass spectra, and elemental analysis, were used to confirm the structures of the synthetic analogues (D1–D20). The presence of stretching bands at 3055–2810 cm^−1^, 3176–2930 cm^−1^, 1750–1640 cm^−1^, and 1567–1480 cm^−1^, in the IR spectrum, provided confirmation of C-H (aliphatic stretching), C-H (aromatic stretching), and C=O, C=C (aromatic stretching band), respectively, in the synthesized analogues.

Moreover, the ^1^H-NMR spectrum confirmed that the synthesized derivatives contained aromatic protons, based on multiplet signals between 6.85 and 7.87 ppm. The singlet(s) signals between 7.49 and 7.97 δ ppm and the presence of -CH = and -CH of thiazolidin-4-one groups were validated in the synthesized derivatives at 2.85–3.25 δ ppm, respectively. Observation of the singlet (s) at 8.30 δ ppm in compound D-11 demonstrated the presence of a proton of the -NH_2_ group. The methoxy group of Ar–OCH_3_ in the compounds D-8, D-11, and D-16 was validated by the presence of singlet(s) in the range of 1.49–3.74 δ ppm.

### 2.1. Antimicrobial Evaluation

A serial tube dilution procedure was used to screen the synthesized 2,5-disubstituted oxadiazole/thiadiazole derivatives for antimicrobial activity (Table 2). In the preliminary screening against Gram-positive (+ve) and Gram-negative (−ve) bacterial strains, compound D-2 showed moderate antibacterial action against *S. aureus*, *E. faecalis*, *E. coli*, and *K. pneumoniae* with MIC 7.55 µM. Similarly, D-6 showed mild antimicrobial action against *S. aureus*, *E. faecalis*, *E. coli*, *K. pneumoniae*, and *T. harzianum* with MIC 7.76 µM. Further screening revealed that compound D-16 was moderately active against *K. pneumoniae* with an MIC*kp* value of 7.82 µM and derivative D-19 was found active against Gram-positive bacterial strains *S. aureus* and *P. aeruginosa* with MIC*sa* = 6.96 µM and MIC*pa* = 6.96 µM. 

Similarly, compound D-17 (MIC*sa* = 7.19 µM) and compound D-20 (MIC*pa* = 6.96 µM) were found to be effective against *S. aureus* and *P. aeruginosa*, respectively. Compound D-12 (MICkp = 8.36 µM) possessed moderate activity against *K. pneumoniae.* Compound D-4 (MIC*e_f_* = 3.58 µM) showed significant antibacterial potential against the *E. faecalis* strain as compared to all derivatives as well as the reference drug. The antifungal screening results revealed that compounds D-4 (MIC*an* = 7.16 µM) and D-15 (MIC*an* = 8.74 µM) had moderate antifungal activity against *A. niger*. Similarly, compounds D-6 and D-11 showed remarkable antifungal potential against *T. harzianum* with MIC*th* = 7.76 µM and 8.48 µM, respectively. Derivative D-20 was found to be most effective against *A. niger* (MIC*an* = 6.90 µM). The antibacterial screening results were compared with those of the standard drug (Amoxicillin (Supplementary Figures S1 and S2)), while the antifungal screening results were compared to those of the standard drug (Fluconazole, (See the Supplementary Figure S3)). Therefore, new antimicrobial agents could be obtained with improved activity by optimizing these synthesized compounds as novel molecules.

### 2.2. MTT Assay Evaluation

**Figure 3:**
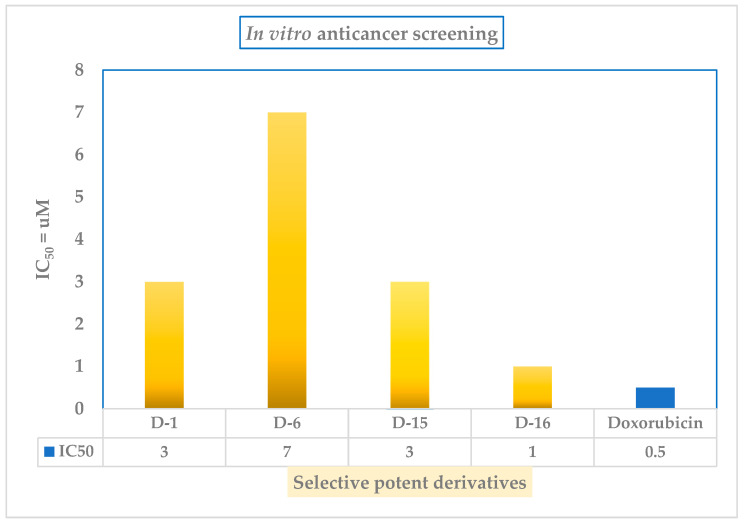
Cytotoxicity screening of potent hiazolidine-4-one derivatives of 1,3,4-oxadiazole/thiadiazole ring.

The anticancer potential of the newly synthesized conjugates was evaluated against human breast cancer using the MCF-7 cell line via the MTT assay. The preliminary screening of the synthesized compounds from the chemical scheme shown in Scheme 1 led to only four derivatives that were further investigated for their anticancer activity against the MCF-7 cell line. Cell viability was measured using the MTT assay at different concentrations and time points. The overall results showed that most of the tested compounds had mild cytotoxic activity (more than 10 µM) towards MCF-7 cells, except four derivatives (1 to 7 μM). Therefore, only four compounds (D-1, D-6, D-15, and D-16) out of the 20 derivatives showed moderate to strong anticancer activity at the range of concentrations of 0.001–0.1 µM (Figure 3). The anticancer activity measured using the MTT assay showed that almost all derivatives exhibited significant anticancer activity as compared to the standard drug. The anticancer activity of all potent derivatives and their IC_50_ values against the MCF-7 cell line are plotted in Figure 3.

### 2.3. Antioxidant Evaluation

**Figure 4:**
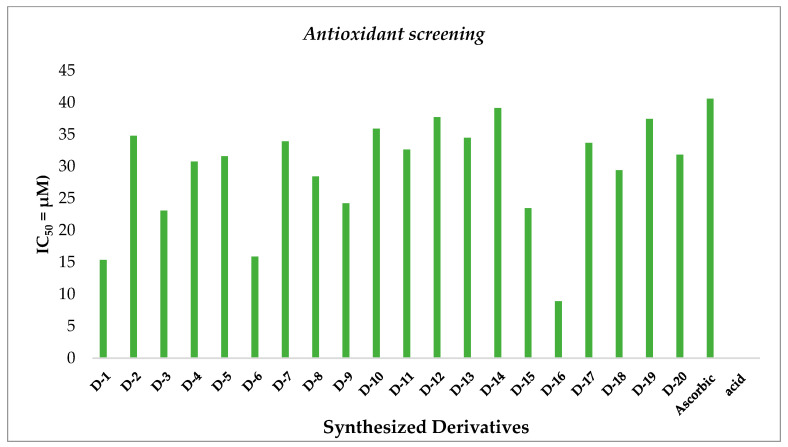
In vitro antioxidant potential of thiazolidin-4-one derivatives of 1,3,4-oxadiazole/thiadiazole ring.

The antioxidant potential of the synthesized 2-substituted-3-(5-substituted-1,3,4-oxadiazol/thiadiazol-2-yl) thiazolidin-4-one derivatives was assessed by the DPPH assay (free radical scavenging method). Ascorbic acid was used as a reference drug to assess the antioxidant properties of the newly synthesized derivatives. An antioxidant assay based on a chain-breaking mechanism, such as DPPH, is one of the most commonly used methods of evaluating a compound’s antioxidant potential. Free radicals such as DPPH can be transformed into diamagnetic molecules by accepting hydrogen or electron radicals from antioxidants [29]. An absorption band at 517 nm is observed for the DPPH solution (methanolic). The color strength of the solution decreases as the DPPH radical reacts with antioxidants/reducing agents to create the bond. Increased antioxidant strength increases the DPPH radical’s ability to absorb electrons, decreasing the intensity of the purple solution to colorless, as measured by spectrophotometry at 517 nm. Calculations were performed for all synthesized molecules to determine their IC_50_ (μg/mL). The antioxidant screening revealed that the synthesized derivatives were more potent than the standard drug. As a result of the antioxidant screening, compound D-16 (IC_50_ = 22.3 µM) was found to be the most potent compound. Table 3 and Figure 4 illustrate the results of the antioxidant evaluation.

### 2.4. Structure–Activity Relationship

**Figure 5:**
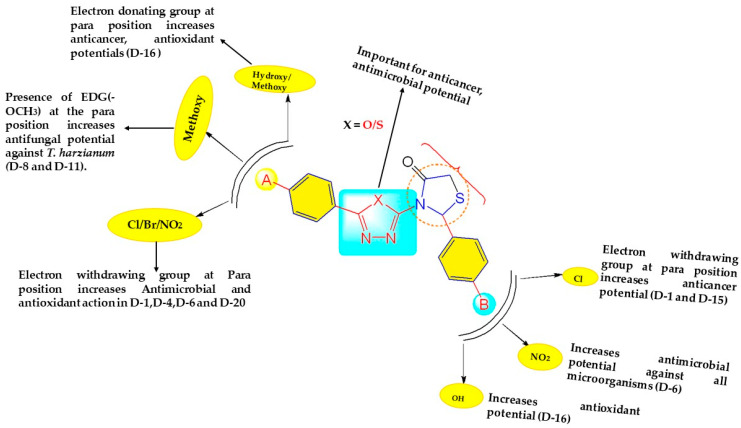
Structure–activity relationship of thiazolidin-4-one derivatives of 1,3,4-oxadiazole/thiadiazole ring.

The following structure–activity relationship can be derived from the evaluations of the anticancer, antimicrobial, and antioxidant activity of the synthesized derivatives (Figure 5).

1. The differences in the substitution and presence of the pharmacophore ring in the final thiazolidin-4-one derivatives of the 1,3,4-oxadiazole/thiadiazole ring played a crucial role in improving the overall biological potential.
2. The presence of an electron-donating group (-OCH_3_/OH) at the para position in the synthesized compound D-16 increased the anticancer and antioxidant potential.
3. The presence of electron-withdrawing groups (Cl/Br) at the para position in the synthesized compound D-4 increased the antimicrobial potential against all Gram-(+ve) and Gram-(−ve) bacterial strains, as well as fungal strains.
4. The electron-withdrawing group (-Cl/Br) at the para position in the synthesized compounds D-1 and D-20 increased the antifungal potential against *Aspergillus niger*.
5. The presence of an electron-donating group (-OCH_3_) at the para position increased the antifungal potential against *Trichoderma harzianum* in the synthesized compounds D-8 and D-11.
6. The electronegative group bromo (Br) in the synthesized compounds D-17 and D-20 enhanced the antibacterial activity against *S. aureus* and *K. pneumoniae*, as well antifungal activity against *A. niger*.
7. Further, these molecules can serve as compounds to create novel antimicrobial and anticytotoxic drugs that are more potent and less toxic.

## 3. Discussion

In this study, 2-substituted-3-(5-substituted-1,3,4 oxadiazol/thiadiazol-2-yl) thiazolidin-4-one derivatives were synthesized from Schiff’s bases by reacting them with thioglycolic acid using a small quantity of zinc chloride as a catalyst. The anticancer potential of the newly synthesized conjugates was evaluated against human breast cancer using the MCF-7 cell line via theMTT assay. The serial tube dilution method was used to evaluate the antimicrobial activity of selected strains of bacteria and fungi against Amoxicillin and Fluconazole as standard drugs. The antioxidant potential was assessed using the stable 2-diphenyl-1-picrylhydrazyl free radical scavenging method. The cytotoxicity screening revealed that conjugates D-1, D-6, D-15, and D-16 possessedhigh efficacy, with an IC_50_ range of 1 to 7 μM, as compared to IC_50_ = 0.5 μM forDoxorubicin as a standard drug. Conjugate D-16, 2-(4-hydroxyphenyl)-3-(5-(4-methoxyphenyl)-1,3,4-thiadiazol-2-yl) thiazolidin-4-one, showed significant anticancer potential against the MCF-7 cancer cell line among all the prepared conjugates. The antimicrobial evaluation indicated that the synthesized derivatives D-2, D-4, D-6, D-19, and D-20 were moderately to significantly active against Gram-(+) and Gram-(−) bacterial strains and fungal strains with MIC ranges of 3.58 µM to 8.74 µM. 

In the antioxidant evaluation, among the synthesized derivatives, D-16 (2-(4-hydroxyphenyl)-3-(5-[4-methoxyphenyl]-1,3,4-thiadiazol-2-yl) thiazolidin-4-one) was found to be the most potent derivative in the series, having IC_50_ = 22.3 µM, as compared toIC_50_ = 111.6 µM for the reference drug ofascorbic acid. Structure–activity relationship (SAR) studies of the synthesized novel derivatives revealed that para-substituted halogen and hydroxy derivatives have remarkable potential against the MCF-7 cancer cell line and antioxidant potential. Similarly, electron-withdrawing groups (Cl/NO_2_) and -donating groups at the para position possess moderate to promising antimicrobialpotential. Therefore, the current evidence indicates that the synthesized derivatives may be promising candidates for use in the prevention and treatment of these infections. These synthesized compounds require further mechanism-based research to understand how they interact with proteins (molecular-level studies).

## 4. Materials and Methods

The derivatives were synthesized without purification using commercially available, analytical-grade chemicals (E. Merck (Darmstadt, Germany) and S. D. Fine Chem. Ltd. (Mumbai, India)). On a melting point apparatus, open glass capillary measurements were performedto determine the melting point (MP). TLC glass plates containing silica gel G were used to monitor each synthetic step using the mobile phases ofethyl acetate:petroleum ether (3:1)* and chloroform:methanol (7:3)**. TMS was used as an internal standard in the NMR measurement of ^1^H spectra on a Bruker Advance III 400 spectrometer. Mass spectra were obtained using Agilent mass spectrometers. A CHN analyzer was used to analyze the elements.

### 4.1. Chemistry

Step 1: Synthesis of substituted 2-amino 1,3,4-oxa/thia(azoles) (03) [30].

A mixture containing substituted carbazide(s) (02) (0.5 moL) and sodium acetate (0.5 moL) was added to water (10 mL) and stirred well; then, substituted aldehydes (01) were added to methanol at room temperature. The stirringwas continued with reduced pressure until the solvent had evaporated completely. Afterward, 1,4-dioxane was added to the residue and the reaction was carried out at 80–85 °C for 4–6 h with the addition of K_2_CO_3_ (1.5 moL) and iodine (0.5 moL).The whole reaction was checked and monitored stepwise by TLC analysis. A mixture of 5% Na_2_S_2_O_3_ (30 mL) was usedafter cooling, and a mixture of CH_2_Cl_2_/MeOH (5:1) was used for extraction. Anhydrous sodium sulfate was used to dry and concentrate the combined organic layer. The given residue was purified through a recrystallization process to obtain the corresponding 2-amino-1,3,4-oxa/thia (azoles) (03) in 80–90% yieldderivatives.

Step 2: General procedure for synthesis of Schiff bases (5).

An ethanolic solution of 03 (0.5 moL) was refluxed with different aromatic aldehydes (4) (0.5 moL). In order to complete the reaction, a small amount of glacial acetic acid (2–3 mL) (dehydrating agent) was added to the whole reaction mixture and the mixture was refluxed for 7–8 h, or until the reaction was complete.Completion of reactions waschecked by TLC. After confirmation of the reaction, the excess ethanol as the solvent was distilled off and the resulting residue mixture was poured onto ice and stirred for 20 min. The resulting precipitates were filtered, washed with ice cold water, dried and recrystallized from ethanol.

Step 3: General procedure for synthesis of 2-substituted-3-(5-substituted-1,3,4-oxa/thiadiazol-2-yl) thiazolidin-4-one (7).

The Schiff base (5) (1 moL) was dissolved in 60 mL of toluene and added to 0.7 mL of thioglycolic acid (6). Dean stark traps were used to reflux the solution. Monitoring of the reaction progress with TLC was carried out. A 3% NaHCO_3_ solution and brine were used to wash the mixture. The resulting organic layer was dried over Na_2_SO_4_ and concentrated under a vacuum. Ethanol was used to recrystallize the products.The all-synthesized derivatives are summarized in terms of their physicochemical properties and spectral analysis in Table 4.

### 4.2. Biological Procedure

#### 4.2.1. MTT Assay

The MTT assay is a cytotoxic test thatmeasures the metabolic activity of cells. It is a colorimetric assay and is based on the reduction of the yellow tetrazolium salt, i.e., MTT (3-(4,5-dimethylthiazol-2-yl)-2,5-diphenyltetrazolium bromide), into a purple formazan product through active mitochondria. The number of active cells present is directly proportional to the total quantity of MTT cleaved, and its quantification is achievedby measuring the absorbance using a colorimeter [31]. This assay was carried out atDeshpande Laboratories (BMG FLUOstar microplate reader), Bhopal (Madhya Pradesh, (India) [32]. The selected compounds to be tested for anticancer activitywere dissolved in DMSO to obtaina range of different concentrations, keeping the concentration of DMSO at <0.1% in all the compounds. Well-maintained MCF-7 breast cancer cells were seeded in 96-well plates. To these wells, test samples of different concentrations were added and they were incubated at 37 °C in a 5% CO_2_ incubator for 96 h. Further, to these wells, MTT reagent was added and they were incubated again for 4 h. Finally, the purple formazan product formedby the cells was collected and dissolved in DMSO (100 μL/mL). Absorption was noted using a colorimeter at 550 nm. Then, the value of percentage inhibition was calculated and plotted against the concentrationon the*X*-axis. Finally, the IC_50_ values were determined [33].

#### 4.2.2. In Vitro Antioxidant Assay

DPPH free radical scavenging assays [34] were used to analyze the antioxidant strength of the thiazolidin-4-one derivatives of the 1,3,4-oxadiazole/thiadiazole ring. In different test tubes, the synthesized conjugates were diluted with methanol to 25, 50, 75 and 100 µg/mL concentrations. Then, 0.039% DPPH was added to each test tube, followed by hard shaking. After wrapping silver foil paper aroundthe test tubes, the mixture was stored in a dark areafor 30 min. The absorbance of the mixtures at 517 nm was measured with a UV–visible double-beam spectrophotometer. Increased antioxidant strength leads to more electrons being taken up by the DPPH radical, decreasing the intensity of the purple solution to colorless, as measured by spectrophotometry at 517 nm. In the data set, IC_50_ values are represented using a minimum of three observations. The IC_50_ value in[µM] was calculated for all the synthesized compounds by using the following equations (Equations (1) and (2)) [34]:

(1) $$Y=Min+\frac{Max-Min}{1\left(\frac{x}{IC_{50}}\right)Hill\;Coefficient}$$

(2) $$\mathrm{Micromolar}\;\left[\mu M\right]=\frac{IC50}{MW}\;\times \;1000$$

#### 4.2.3. In Vitro Antimicrobial Assay

The serial tube dilution method [35] was used to evaluate the antimicrobial potential of the synthesized compounds using Fluconazole (antifungal) and Amoxicillin (antibacterial) as standard drugs. This study used Gram-positive (+ve) (MTCC-3160 (*S. aureus*), (MTCC-441 (*E. faecalis*)) and Gram-negative (−ve) (MTCC-3541 (*P. aeruginosa*), (MTCC-443 (*E. coli*) and MTCC-9024 (*K. pneumoniae*)) bacteria. In this study, the MTCC-3683 (*T. harzianum*) and MTCC-281 (*A. niger*) strains were tested for their antifungal potential. The double-strength nutrient broth I.P. (for bacteria) or sabouraud dextrose broth I.P. (for fungi) wasused to test their antimicrobial activity [36]. A dimethyl sulfoxide stock solution was prepared for the test and standard drugs. Moreover, dimethyl sulfoxide was added to the test medium as a control set at the same dilutions. Results were recorded as theMIC after incubating the samples at 25 ± 1 °C (7 days) for *A. niger*, at 25 ± 1 °C (36 h) for *T. harzianum* and at 37 ± 1 °C (24 h) for bacteria (s), respectively. The MIC for the tested compound was observed as the lowest concentration of the compound that prevented microorganism growth inside the test tube [37]. The antimicrobial potential screening value was calculated as amicromolar value [µM] for all the synthesized compounds by using the following equation (Equation (3)):

(3) $$\mathrm{Micromolar}\;\left[\mu M\right]=\frac{MIC}{MW}\;\times \;1000$$

## 5. Conclusions

A novel series of thiazolidin-4-one derivatives of 1,3,4-oxadiazole/thiadiazole rings are described in this paper. The cytotoxicity screening indicated that conjugates D-1, D-6, D-15, and D-16 had a high level of effectiveness, with an IC_50_ range of 1 to 7 μM, as opposed to IC_50_ = 0.5 μM for Doxorubicin as the benchmark drug. In this work, 2-(4-hydroxyphenyl)-3-(5-(4-methoxyphenyl)-1,3,4-thiadiazol-2-yl) thiazolidin-4-one (D-16) showed significant anticancer potential (IC_50_ = 1 μM) against the MCF-7 cancer cell line and also showed significant antioxidant potential (IC_50_ = 8.90 μg/mL) among all the prepared derivatives. Antimicrobial activity was observed in all newly synthesized compounds. A study of the structure–activity relationships (SARs) of the synthesized novel derivatives showed that para-substituted halogens and hydroxyl derivatives were highly effective against MCF-7 cancer cells and were powerful antioxidants. In a similar way, electron-withdrawing groups (Cl/NO_2_) and -donating groups at the para position were moderately to highly antimicrobial.

Using the insights gained from this study, novel molecules could be proposed that may have anticancer activity and antimicrobial or antioxidant potential. This could lead to the development of new drugs or treatments that may improve patient outcomes. Additionally, the findings of this study may be used to identify other potential compounds that may have similar properties or activity. While initial efforts focused on the development of anticytotoxic agents for cancer treatment, new cancer biomarkers and new antimicrobials have led to the discovery of compounds with improved safety profiles. In addition, more research is needed to further investigate the mechanisms of action of the novel compounds and to develop new strategies for drug delivery and targeting.

## Figures and Tables

**Table 1:** Different substitutions of Scheme 1 (thiazolidin-4-one derivatives of 1,3,4-oxadiazole/thiadiazole ring).

S. No.	Derivative (5)	Y (2)	X	R_1_	R_2_	R_3_	R_4_
1.	D-1	O	O	NO_2_	Cl	H	H
2.	D-2	O	O	NO_2_	NO_2_	H	H
3.	D-3	O	O	NO_2_	OH	H	H
4.	D-4	O	O	Cl	Br	H	H
5.	D-5	O	O	Cl	OCH_3_	H	H
6.	D-6	O	O	Cl	NO_2_	H	H
7.	D-7	O	O	Cl	Cl	H	H
8.	D-8	O	O	OCH_3_	OCH_3_	H	H
9.	D-9	O	O	OCH_3_	H	Cl	Cl
10.	D-10	O	O	OCH_3_	NO_2_	H	H
11.	D-11	O	O	OCH_3_	NH_2_	H	H
12.	D-12	O	O	Cl	OH	H	H
13.	D-13	O	O	Cl	NH_2_	H	H
14.	D-14	O	O	NO_2_	I	H	H
15.	D-15	O	O	Cl	H	H	H
16.	D-16	S	S	OCH_3_	OH	H	H
17.	D-17	S	S	Br	OH	H	H
18.	D-18	S	S	OCH_3_	OCH_3_	H	H
19.	D-19	S	S	OCH_3_	Br	H	H
20.	D-20	S	S	Br	Cl	H	H

**Table 2:** Antimicrobial screening of thiazolidin-4-one derivatives of 1,3,4-oxadiazole/thiadiazole ring.

Antimicrobial Screening (MIC = µM)							
Derivative	Antibacterial Screening					Antifungal Screening	
	SA	EF	PA	EC	KP	TH	AN
D-1	31.09	15.55	15.55	31.09	15.55	15.55	31.09
D-2	7.55	7.55	15.13	7.55	7.55	15.13	15.13
D-3	16.28	32.55	16.28	32.55	32.55	16.28	32.55
D-4	7.16	3.58	7.16	7.16	7.16	14.33	7.16
D-5	32.30	32.30	16.15	16.15	16.15	32.30	64.60
D-6	7.76	7.76	7.76	15.55	7.76	7.76	31.09
D-7	31.89	31.89	31.89	31.89	31.89	15.94	31.89
D-8	32.64	32.64	32.64	32.64	32.64	8.15	32.64
D-9	59.24	29.62	29.62	59.24	14.81	29.62	29.62
D-10	15.70	15.70	15.70	15.70	15.70	31.41	15.70
D-11	16.98	16.98	16.98	16.98	16.98	8.48	16.98
D-12	16.76	67.02	33.51	33.51	8.36	33.51	33.51
D-13	33.60	67.20	33.60	33.60	33.60	33.60	67.20
D-14	50.61	50.61	50.61	50.61	25.30	25.30	25.30
D-15	70.03	35.01	35.01	35.01	35.01	35.01	8.74
D-16	15.66	31.33	15.66	15.66	7.82	15.66	31.33
D-17	7.19	14.40	14.40	28.80	7.19	28.80	28.80
D-18	7.82	15.66	31.33	15.66	15.66	31.33	31.33
D-19	6.96	13.95	6.96	13.95	13.95	13.95	27.90
D-20	13.83	27.65	6.90	13.83	27.65	27.65	6.90
*Amoxicillin*	4.29 *	4.29 *	4.29 *	4.29 *	4.29 *		
*Fluconazole*						5.10 **	5.10 **

SA: *Staphylococcus aureus*, EF: *Enterococcus faecalis*, PA: *Pseudomonas aeruginosa*, EC: *Escherichia coli*, KP: *Klebsiella pneumoniae*, TH: *Trichoderma harzianum*, AN: *Aspergillus Niger*. **Positive control**—*Amoxicillin* *, *Fluconazole* **.

**Table 3:** In vitro antioxidant activity of thiazolidin-4-one derivatives of 1,3,4-oxadiazole/thiadiazole ring.

Derivative	IC_50_ (µM)
D-1	38.3
D-2	84.3
D-3	60.2
D-4	70.6
D-5	81.6
D-6	39.5
D-7	86.6
D-8	74.2
D-9	57.4
D-10	90.2
D-11	88.7
D-12	101.1
D-13	92.7
D-14	79.2
D-15	65.7
D-16	22.3
D-17	77.6
D-18	73.7
D-19	83.5
D-20	70.5
Ascorbic acid	111.6

**Table 4:** Physicochemical and spectral characteristics of 1,3,4 oxadiazole/thiadiazoles.

Derivative	Physicochemical and Spectral Characteristics
D-1	2-(4-chlorophenyl)-3-(5-(4-nitrophenyl)-1,3,4-oxadiazol-2-yl) thiazolidin-4-one. Light yellowish color, M.pt. 180–182 °C, R*f* value: 0.7*%, yield: 37%. Chemical formula: C_17_H_11_ClN_4_O_4_S. Molecular weight: 402. IR (KBr pellets) cm^−1^: 2926.14 (C-H str., aliphatic), 1732.18 (C=O str., thiazolidine ring), 1678.53 (C=N str., imine group), 1427.75 (C=C str., aromatic ring), 1602.13 (C=C str., methylene group), 1317.75 (C-N str., thiazolidine ring), 754.46 (C-Cl str., aromatic ring). ^1^H NMR (δ, DMSO):1.62–1.66 (m, 2H, 1,3-thiazolidin-4-one), 2.85–2.89 (m, 1H, 1,3-thiazolidin-4-one), 7.55–7.62 (m, 4H, aromatic H, incorporated with 1,3-thiazolidin-4-one) 7.85–7.87 (m, 4H, aromatic H, incorporated with 1,3,4-oxadiazole). *m*/*z*: 402.700. Elemental analysis (CHN) theoretical calc: C, 50.69; H, 2.75; N, 13.91; found: C, 50.60; H, 2.79; N, 13.96.
D-2	2-(4-nitrophenyl)-3-(5-(4-nitrophenyl)-1,3,4-oxadiazol-2-yl)thiazolidin-4-one. Orange color, M.pt. 175–177 °C, R*f* value: 0.8*%, yield: 58%. Chemical formula: C_17_H_11_N_5_O_6_S. Molecular weight: 413. IR (KBr pellets) cm^−1^: 2984.07 (C-H str., aliphatic), 3070.31 (C-H str., aromatic), 1728.84 (C=O str., thiazolidine ring), 1650.74 (C=N str., imine group), 1464.21 (C=C str., aromatic ring), 1650.74 (C=C str., methylene group), 1500.11 (N-O str., nitro group), 1385.83 (C-N str., thiazolidine ring), 653.79 (C-S bend., thiazolidine ring). ^1^H NMR (δ, DMSO):2.15–2.29 (m, 2H, 1,3-thiazolidin-4-one), 3.06–3.25 (m, 1H, 1,3-thiazolidin-4-one), 7.21–7.22 (m, 4H, aromatic H, incorporated with 1,3-thiazolidin-4-one) 7.43–7.52 (m, 4H, aromatic H, incorporated with 1,3,4-oxadiazole). *m*/*z*: 413.04. Elemental analysis (CHN) theoretical calc: C, 49.40; H, 2.68; N, 16.94; found: C, 49.50; H, 2.54; N, 16.98.
D-3	2-(4-hydroxyphenyl)-3-(5-(4-nitrophenyl)-1,3,4-oxadiazol-2-yl)thiazolidin-4-one. White color, M.pt. 165–167 °C, R*f* value: 0.7*%, yield: 44%. Chemical formula: C_17_H_12_N_4_O_5_S. Molecular weight: 384. IR (KBr pellets) cm^−1^: 2962.12 (C-H str., aliphatic), 3243.04 (C-H str., aromatic), 1688.01 (C=O str., thiazolidine ring), 1634.76 (C=N str., imine group), 1431.79 (C=C str., aromatic ring), 1597.91 (C=C str., methylene group), 1502.35 (N-O str., nitro group), 1371.40 (C-N str., thiazolidine ring), 1315.68 (OH str., aromatic ring), 673.86 (C-S bend., thiazolidine ring). ^1^H NMR (δ, DMSO):1.82–1.85 (m, 2H, 1,3-thiazolidin-4-one), 2.87–2.88 (m, 1H, 1,3-thiazolidin-4-one), 7.55–7.61 (m, 4H, aromatic H, incorporated with 1,3-thiazolidin-4-one) 7.85–7.87 (m, 4H, aromatic H, incorporated with 1,3,4-oxadiazole), 8.64 (s, 1H, aromatic OH). *m*/*z*: 384.05. Elemental analysis (CHN) theoretical calc: C, 53.12; H, 3.15; N, 14.58. Found: C, 53.02; H, 3.20; N, 14.63.
D-4	2-(4-bromophenyl)-3-(5-(4-chlorophenyl)-1,3,4-oxadiazol-2-yl)thiazolidin-4-one.White color, M.pt. 183–185 °C, R*f* value: 0.7*%, yield: 49%. Chemical formula: C_17_H_11_BrClN_3_O_2_S. Molecular weight: 436. IR (KBr pellets) cm^−1^: 2986.68 (C-H str., aliphatic), 3041.69 (C-H str., aromatic), 1643.93 (C=O str., thiazolidine ring), 1426.27 (C=C str., aromatic ring), 1530.61 (C=C str., methylene group), 1340.36 (C-N str., thiazolidine ring), 596.93 (C-Br str., aromatic ring), 754.99 (C-Cl str., aromatic ring), 696.93 (C-S bend., thiazolidine ring), 710.85 (C-S bend., thiazolidine ring). ^1^H NMR (δ, DMSO):2.15–2.29 (m, 2H, 1,3-thiazolidin-4-one), 3.06–3.23 (m, 1H, 1,3-thiazolidin-4-one), 6.85–6.86 (m, 4H, aromatic H, incorporated with 1,3-thiazolidin-4-one) 7.43–7.51 (m, 4H, aromatic H, incorporated with 1,3,4-oxadiazole). *m*/*z*: 436.800. Elemental analysis (CHN) theoretical calc: C, 46.76; H, 2.54; N, 9.62. Found: C, 46.72; H, 2.60; N, 9.66.
D-5	3-(5-(4-chlorophenyl)-1,3,4-oxadiazol-2-yl)-2-(4-methoxyphenyl)thiazolidin-4-one.Green color, M.pt. 159–161 °C, R*f* value: 0.8**%, yield: 61%. Chemical formula: C_18_H_14_ClN_3_O_3_S. Molecular weight: 387. IR (KBr pellets) cm^−1^: 2931.36 (C-H str., aliphatic of methoxy), 3381.33 (C-H str., aromatic), 1628.57 (C=O str., thiazolidine ring), 1427.89 (C=C str., aromatic ring), 1279.22 (C-N str., thiazolidine ring), 710.85 (C-Cl str., aromatic ring). ^1^H NMR (δ, DMSO):1.10 (s, 3H, OCH_3_, methoxyphenyl incorporated with 1,3,4-oxadiazole),1.72–1.74 (m, 2H, 1,3-thiazolidin-4-one), 2.51–2.53 (m, 1H, 1,3-thiazolidin-4-one), 4.54–4.55 (m, 4H, aromatic H, incorporated with 1,3-thiazolidin-4-one) 4.91–4.92 (m, 4H, aromatic H, incorporated with 1,3,4-oxadiazole). *m*/*z*: 387.700. Elemental analysis (CHN) theoretical calc: C, 55.74; H, 3.64; N, 10.83. Found: C, 55.54; H, 3.80; N, 10.87.
D-6	3-(5-(4-chlorophenyl)-1,3,4-oxadiazol-2-yl)-2-(4-nitrophenyl)thiazolidin-4-one. Orange color, M.pt. 199–201 °C, R*f* value: 0.6*%, yield: 57%. Chemical formula: C_17_H_11_ClN_4_O_4_S, Molecular Weight: 402. IR (KBr pellets) cm^−1^: 3005.81 (C-H str., aromatic), 1604.43 (C=O str., thiazolidine ring), 1454.67 (C=C str., aromatic ring), 1513.94 (N-O str., nitro group), 1272.88 (C-N str., thiazolidine ring), 808.73 (C-Cl str., aromatic ring), 559.36 (C-S bend., thiazolidine ring). ^1^H NMR (δ, DMSO):2.15–2.29 (m, 2H, 1,3-thiazolidin-4-one), 3.06–3.23 (m, 1H, 1,3-thiazolidin-4-one), 6.85–7.01 (m, 4H, aromatic H, incorporated with 1,3-thiazolidin-4-one), 7.21–7.62 (m, 4H, aromatic H, incorporated with 1,3,4-oxadiazole). *m*/*z*: 402.020. Elemental analysis (CHN) theoretical calc: C, 50.69; H, 2.75; N, 13.91. Found: C, 50.75; H, 2.55; N, 14.05.
D-7	2-(4-chlorophenyl)-3-(5-(4-chlorophenyl)-1,3,4-oxadiazol-2-yl)thiazolidin-4-one. Brownish color, M.pt. 212–214°C, R*f* value: 0.7*%: yield 32%. Chemical formula: C_17_H_11_C_l2_N_3_O_2_S. Molecular weight: 392. IR (KBr pellets) cm^−1^: 3081.12 (C-H str., aromatic), 1661.11 (C=O str., thiazolidine ring), 1584.02 (C=C str., methylene group), 1499.76 (C=C str., aromatic ring), 1381.30 (C-N str., thiazolidine ring), 750.45 (C-Cl str., aromatic ring). ^1^H NMR (δ, DMSO):1.82–1.85 (m, 2H, 1,3-thiazolidin-4-one), 2.85–2.89 (m, 1H, 1,3-thiazolidin-4-one), 7.55–7.60 (m, 4H, aromatic H, incorporated with 1,3-thiazolidin-4-one), 7.85–7.87 (m, 4H, aromatic H, incorporated with 1,3,4-oxadiazole). *m*/*z*: 392.600. Elemental analysis (CHN) theoretical calc: C, 52.05; H, 2.83; N, 10.71. Found: C, 52.25; H, 2.70; N, 10.64.
D-8	2-(4-methoxyphenyl)-3-(5-(4-methoxyphenyl)-1,3,4-oxadiazol-2-yl) thiazolidin-4-one. Cream color, M.pt. 220–222 °C, R*f* value: 0.7*%, yield: 72%. Chemical formula: C_19_H_17_N_3_O_4_S. Molecular weight: 383. IR (KBr pellets) cm^−1^: 2933.62 (C-H str., aliphatic), 3112.44 (C-H str., aromatic), 1663.37 (C=O str., thiazolidine ring), 1447.61 (C=C str., aromatic ring), 1481.90 (C=C str., methylene group), 1324.01 (C-N str., thiazolidine ring), 656.78 (C-S bend., thiazolidine ring). ^1^H NMR (δ, DMSO): 0.81 (s, 3H, OCH_3_, methoxyphenyl incorporated with 1,3-thiazolidin-4-one), 1.28 (s, 3H, OCH_3_, methoxyphenyl incorporated with 1,3,4-oxadiazole), 2.15–2.29 (m, 2H, 1,3-thiazolidin-4-one), 3.06–3.23 (m, 1H, 1,3-thiazolidin-4-one), 6.85–7.01 (m, 4H, aromatic H, incorporated with 1,3-thiazolidin-4-one), 7.22–7.62 (m, 4H, aromatic H, incorporated with 1,3,4-oxadiazole). *m*/*z*: 383.600. Elemental analysis (CHN) theoretical calc: C, 59.52; H, 4.47; N, 10.96. Found: C, 59.42; H, 4.50; N, 11.03.
D-9	2-(3,5-dichlorophenyl)-3-(5-(4-methoxyphenyl)-1,3,4-oxadiazol-2-yl)thiazolidin-4-one. White color, M.pt. 213–215 °C, R*f* value: 0.8*%, yield: 63%. Chemical formula: C_18_H_13_Cl_2_N_3_O_3_S. Molecular weight: 422. IR (KBr pellets) cm^−1^: 3027.55 (C-H str., aromatic), 1673.58 (C=O str., thiazolidine ring), 1419.27 (C=C str., aromatic ring), 1485.15 (C=C str., methylene group), 1301.04 (C-N str., thiazolidine ring). 752.18 (C-Cl str., aromatic ring), 657.56 (C-S bend., thiazolidine ring). ^1^H NMR (δ, DMSO): 0.97 (s, 3H, OCH_3_, methoxyphenyl incorporated with 1,3-thiazolidin-4-one), 1.82–1.85 (m, 2H, 1,3-thiazolidin-4-one), 4.53–4.54 (m, 1H, 1,3-thiazolidin-4-one), 4.55–4.63 (m, 4H, aromatic H, incorporated with 1,3-thiazolidin-4-one), 4.64–4.93 (m, 4H, aromatic H, incorporated with 1,3,4-oxadiazole). *m*/*z*: 423.010. Elemental analysis (CHN) theoretical calc: C, 51.20; H, 3.10; N, 9.95. Found: C, 51.22; H, 3.12; N, 9.99.
D-10	3-(5-(4-methoxyphenyl)-1,3,4-oxadiazol-2-yl)-2-(4-nitrophenyl)thiazolidin-4-one. Pale yellowish color, M.pt. 207–209 °C, R*f* value: 0.8*%, yield: 67%. Chemical formula: C_18_H_14_N_4_O_5_S. Molecular weight: 398. IR (KBr pellets) cm^−1^: 3088.63 (C-H str., aromatic), 1632.03 (C=O str., thiazolidine ring), 1515.85 (N-O str., nitro group), 1462.27 (C-H str., methoxy group), 1224.18 (C-N str., thiazolidine ring), 697.83 (C-S bend., thiazolidine ring). ^1^H NMR (δ, DMSO): δ 1.23 (s, 3H, OCH_3_, methoxyphenyl incorporated with 1,3-thiazolidin-4-one), 2.27–2.29 (m, 2H, 1,3-thiazolidin-4-one), 3.11–3.23 (m, 1H, 1,3-thiazolidin-4-one), 4.54–4.55 (m, 4H, aromatic H, incorporated with 1,3-thiazolidin-4-one), 4.91–4.92 (m, 4H, aromatic H, incorporated with 1,3,4-oxadiazole). *m*/*z*: 398.600. Elemental analysis (CHN) theoretical calc: C, 54.27; H, 3.54; N, 14.06. Found: C, 54.30; H, 3.51; N, 14.12.
D-11	2-(4-aminophenyl)-3-(5-(4-methoxyphenyl)-1,3,4-oxadiazol-2-yl)thiazolidin-4-one.Light green color, M.pt. 178–180 °C, R*f* value: 0.6**%, yield: 49%. Chemical formula: C_18_H_16_N_4_O_3_S. Molecular weight: 368. IR (KBr pellets) cm^−1^: 2983.44 (C-H str., aliphatic), 3222.31 (C-H str., aromatic), 1686.04 (C=O str., thiazolidine ring), 1634.63 (N-H str., amine group), 1281.36 (C-N str., aromatic amine), 1122.07 (C-O str., oxadiazole ring), 700.14 (C-S bend., thiazolidine ring). ^1^H NMR (δ, DMSO): 1.49 (s, 3H, OCH_3_, methoxyphenyl incorporated with 1,3-thiazolidin-4-one), 2.85–2.89 (m, 2H, 1,3-thiazolidin-4-one), 4.53–4.55 (m, 1H, 1,3-thiazolidin-4-one), 4.62–4.65 (m, 4H, aromatic H, incorporated with 1,3-thiazolidin-4-one), 4.90–4.93 (m, 4H, aromatic H, incorporated with 1,3,4-oxadiazole), 8.30 (s, 2H, aromaticNH_2_). *m*/*z*: 368.120. Elemental analysis (CHN) theoretical calc: C, 58.68; H, 4.38; N, 15.21. Found: C, 58.08; H, 4.38; N, 15.81.
D-12	3-(5-(4-chlorophenyl)-1,3,4-oxadiazol-2-yl)-2-(4-hydroxyphenyl)thiazolidin-4-one. Green color, M.pt. 179–181 °C, R*f* value: 0.7*%, yield 34%. Chemical formula: C_17_H_12_ClN_3_O_3_S. Molecular weight: 373. IR (KBr pellets) cm^−1^: 2971.65 (C-H str., aliphatic), 3230.37 (C-H str., aromatic), 1667.67 (C=O str., thiazolidine ring), 1628.07 (C=N str., imine group), 1440.28 (C=C str., aromatic ring), 1522.71 (C=C str., methylene group), 1291.32 (C-N str., thiazolidine ring), 1315.76 (OH str., aromatic ring), 770.93 (C-Cl str., aromatic ring), 697.66 (C-S bend., thiazolidine ring). ^1^H NMR (δ, DMSO): 2.15–2.28 (m, 2H, 1,3-thiazolidin-4-one), 3.11–3.23 (m, 1H, 1,3-thiazolidin-4-one), 6.85–6.86 (m, 4H, aromatic H, incorporated with 1,3-thiazolidin-4-one), 7.22–7.52 (m, 4H, aromatic H, incorporated with 1,3,4-oxadiazole), 9.43 (s, 1H, aromatic OH). *m*/*z*: 373.010. Elemental analysis (CHN) theoretical calc: C, 54.62; H, 3.24; N, 11.24. Found: C, 54.52; H, 3.20; N, 11.38.
D-13	2-(4-aminophenyl)-3-(5-(4-chlorophenyl)-1,3,4-oxadiazol-2-yl)thiazolidin-4-one.Yellowish, M.pt. 214–216 °C, R*f* value: 0.6**%,yield: 56%. Chemical formula: C_17_H_13_ClN_4_O_2_S. Molecular weight: 372. IR (KBr pellets) cm^−1^: 3080.17 (C-H str., aromatic), 1661.01 (C=O str., thiazolidine ring), 1601.66 (N-H str., amine group), 1281.36 (C-N str., aromatic amine), 1132.84 (C-O str., oxadiazole ring). 750.99 (C-Cl str., aromatic ring). ^1^H NMR (δ, DMSO): 3.16–4.11 (m, 2H, 1,3-thiazolidin-4-one), 6.20 (s, 1H, 1,3-thiazolidin-4-one), 6.71–6.7 (m, 4H, aromatic H, incorporated with 1,3-thiazolidin-4-one), 7.8–8.3 (m, 4H, aromatic H, incorporated with 1,3,4-oxadiazole). *m*/*z*: 372. Elemental analysis (CHN) theoretical calc: C, 54.77; H, 3.51; N, 15.03. Found: C, 54.78; H, 3.60; N, 14.93.
D-14	2-(4-iodophenyl)-3-(5-(4-nitrophenyl)-1,3,4-oxadiazol-2-yl)thiazolidin-4-one.Reddish color, M.pt. 231–233 °C, R*f* value: 0.7*%, yield: 64%. Chemical formula: C_17_H_11_IN_4_O_4_S. Molecular weight: 494. IR (KBr pellets) cm^−1^: 3143.30 (C-H str., aromatic), 1641.59 (C=O str., thiazolidine ring), 1596.32 (C=N str., imine group), 1464.32 (C=C str., aromatic ring), 1541.32 (C=C str., methylene group), 1263.59 (C-N str., thiazolidine ring), 1315.76 (OH str., aromatic ring), 622.37 (C-S bend., thiazolidine ring), 525.84 (C-I str., *para*position on aromatic ring). ^1^H NMR (δ, DMSO):3.66–4.13 (m, 2H, 1,3-thiazolidin-4-one), 6.38 (s, 1H, 1,3-thiazolidin-4-one), 7.27–7.28 (m, 4H, aromatic H, incorporated with 1,3-thiazolidin-4-one), 7.88–8.32 (m, 4H, aromatic H, incorporated with 1,3,4-oxadiazole). *m*/*z*: 493. Elemental analysis (CHN) theoretical calc: C, 41.31; H, 2.24; N, 11.34. Found: C, 41.28; H, 2.28; N, 11.35.
D-15	3-(5-(4-chlorophenyl)-1,3,4-oxadiazol-2-yl)-2-phenylthiazolidin-4-one.Brown color, M.pt. 217–219 °C, R*f* value: 0.7*%, yield: 69%. Chemical formula: C_17_H_12_ClN_3_O_2_S. Molecular weight: 357. IR (KBr pellets) cm^−1^: 1735.50 (C=O str., thiazolidine ring), 1607.87 (N-H str., amine group), 1281.38 (C-N str., aromatic amine), 1173.11 (C-O str., oxadiazole ring). 1422.61 (C=C str., aromatic ring), 633.81 (C-S bend., thiazolidine ring). ^1^H NMR (δ, DMSO):3.70–4.10 (m, 2H, 1,3-thiazolidin-4-one), 6.18 (s, 1H, 1,3-thiazolidin-4-one), 7.32–7.47 (m, 5H, aromatic H, incorporated with 1,3-thiazolidin-4-one), 7.77–7.86 (m, 4H, aromatic H, incorporated with 1,3,4-oxadiazole). *m*/*z*: 357. Elemental analysis (CHN) theoretical calc: C, 57.07; H, 3.38; N, 11.74. Found: C, 57.17; H, 3.26; N, 11.76.
D-16	2-(4-hydroxyphenyl)-3-(5-(4-methoxyphenyl)-1,3,4-thiadiazol-2-yl)thiazolidin-4-one. Pale yellowish color, M.pt. 170–172 °C, R*f* value: 0.6**%, yield: 43%. Chemical formula: C_18_H_15_N_3_O_3_S_2._Molecular weight: 385. IR (KBr pellets) cm^−1^: 2993.78 (C-H str., aliphatic), 3040.45 (C-H str., aromatic), 1600.15 (C=O str., thiazolidine ring), 1474.64 (C=C str. aromatic ring), 1520.64 (C=C str., methylene group), 1338.03 (C-N str., thiazolidine ring), 650.39 (C-S bend., thiazolidine ring). ^1^H NMR (δ, DMSO): *3.82* (s, 3H, OCH_3_, methoxyphenyl incorporated with 1,3,4-thiadiazole), 3.58–3.66 (m, 2H, 1,3-thiazolidin-4-one), 6.33 (s, 1H, OH, incorporated with benzene), 6.74–7.76 (m, 2H, aromatic H, incorporated with 1,3-thiazolidin-4-one), 7.00–7.01 (m, 2H, aromatic H, incorporated with 1,3,4-oxadiazole), 7.063–7.078 (m, 4H, aromatic H, incorporated with 1,3,4-oxadiazole). *m*/*z*: 384.700. Elemental analysis (CHN) theoretical calc: 57.13; H, 4.29; N, 10.52. Found: C, 57.09; H, 4.27; N, 10.58.
D-17	3-(5-(4-bromophenyl)-1,3,4-thiadiazol-2-yl)-2-(4-hydroxyphenyl) thiazolidin-4-one.Light green, M.pt. 191–193°C, R*f* value: 0.6**%,yield: 51%. Chemical formula: C_17_H_12_BrN_3_O_2_S_2._Molecular weight: 434. IR (KBr pellets) cm^−1^: 1645.68 (C=O str., thiazolidine ring), 1456.99 (C=C str. aromatic ring), 1511.08 (C=C str., methylene group), 1246.43 (C-N str., thiazolidine ring), 1370.45 (OH str., aromatic ring), 665.42 (C-Br str., aromatic ring), 513.53 (C-S bend., thiazolidine ring). ^1^H NMR (δ, DMSO):3.63–3.67 (m, 2H, 1,3-thiazolidin-4-one), 3.54–3.61 (S, 1H, 1,3-thiazolidin-4-one), 6.67–6.77 (d, 4H, aromatic H, incorporated with 1,3-thiazolidin-4-one), 7.05–7.74 (d, 4H, aromatic H, incorporated with 1,3,4-oxadiazole), 7.57–7.59 (d, 2H, aromatic OH). *m*/*z*: 434.950. Elemental analysis (CHN) theoretical calc:C, 47.01; H, 2.79; N, 9.67. Found: C, 47.13; H, 2.70; N, 9.64.
D-18	2-(4-methoxyphenyl)-3-(5-(4-methoxyphenyl)-1,3,4-thiadiazol-2-yl) thiazolidin-4-one.White color, M.pt. 224–226 °C, R*f* value: 0.6*%, yield: 57%. Chemical formula: C_19_H_17_N_3_O_3_S_2_. Molecular weight: 399. IR (KBr pellets) cm^−1^: 1630.66 (C=O str., thiazolidine ring), 1441.03 (C=C str. aromatic ring), 1533.52 (C=C str., methylene group), 1247.22 (C-N str., thiazolidine ring), 1209.10, 1355.60 (OH str., aromatic ring), 671.65 (C-S bend., thiazolidine ring). ^1^H NMR (δ, DMSO):3.79–3.80 (s, 3H, OCH_3_, methoxyphenyl incorporated with 1,3-thiazolidin-4-one), 3.60–3.67 (t, 2H, 1,3-thiazolidin-4-one), 6.42 (s, 1H, 1,3-thiazolidin-4-one), 3.79–3.80 (s, 3H, OCH_3_, methoxyphenyl incorporated with benzene ring), 6.87–6.89 (d, 4H, aromatic H, incorporated with 1,3-thiazolidin-4-one), 7.00–7.02 (m, 4H, aromatic H, incorporated with 1,3,4-oxadiazole). *m*/*z*: 399.070. Elemental analysis (CHN) theoretical calc: C, 57.13; H, 4.29; N, 10.52. Found: C, 57.01; H, 4.10; N, 11.10.
D-19	2-(4-bromophenyl)-3-(5-(4-methoxyphenyl)-1,3,4-thiadiazol-2-yl)thiazolidin-4-one.White color, M.pt. 236–238 °C, R*f* value: 0.8*%, yield: 58%. Chemical formula: C_18_H_14_BrN_3_O_2_S_2_. Molecular weight: 448. IR (KBr pellets) cm^−1^: 2919.56 (C-H str., aliphatic), 1734.97 (C=O str., thiazolidine ring), 1463.83 (C=C str. aromatic ring), 1539.41 (C=C str., methylene group), 1335.34 (C-N str., thiazolidine ring), 1539.41 (C=N str., oxadiazole ring), 621.59 (C-S bend., thiazolidine ring), 586.59 (C-Br str., aromatic ring). ^1^H NMR (δ, DMSO):3.80(m, 3H, OCH_3_, methoxyphenyl incorporated with 1,3,4-oxadiazole), 3.54–3.60 (m, 2H, 1,3-thiazolidin-4-one), 6.41 (s, 1H, 1,3-thiazolidin-4-one), 7.38–7.48 (m, 4H, aromatic H, incorporated with 1,3-thiazolidin-4-one), 6.99–7.14 (m, 4H, aromatic H, incorporated with 1,3,4-oxadiazole). *m*/*z*: 440.600. Elemental analysis (CHN) theoretical calc: C, 48.22; H, 3.15; N, 9.37. Found: C, 48.42; H, 3.01; N, 9.31.
D-20	3-(5-(4-bromophenyl)-1,3,4-thiadiazol-2-yl)-2-(4-chlorophenyl)thiazolidin-4-one.Brown color, M.pt. 247–249 °C, R*f* value: 0.7*%, yield: 72%. Chemical formula: C_17_H_11_BrClN_3_OS_2._Molecular weight: 452. IR (KBr pellets) cm^−1^: 2922.29 (C-H str., aliphatic), 1737.33 (C=O str., thiazolidine ring), 1460.08 (C=C str. aromatic ring), 1368.40 (C-N str., thiazolidine ring), 1539.41 (C=N str., oxadiazole ring), 753.28 (C-Cl str., *para*position on aromatic ring), 551.85 (C-Br str., aromatic ring), 673.71 (C-S bend., thiazolidine ring). ^1^H NMR (δ, DMSO): 3.58–3.66 (m, 2H, 1,3-thiazolidin-4-one), 6.46 (s, 1H, 1,3-thiazolidin-4-one), 7.15–7.29 (m, 4H, aromatic H, incorporated with 1,3-thiazolidin-4-one), 7.40–7.59 (m, 4H, aromatic H, incorporated with 1,3,4-oxadiazole). *m*/*z*: 441.720. Elemental analysis (CHN) theoretical calc: C, 45.10; H, 2.45; N, 9.28. Found: C, 45.15; H, 2.42; N, 9.22.
TLC mobile phase (R*f*)	“Ethyl acetate:petroleum ether (3:1)” *, “chloroform:methanol (7:3)” **

## Data Availability

Data is contained within the article.

## Supplementary Materials

The following are available online at https://www.mdpi.com/article/10.3390/ph16060805/s1, Figure S1: Antibacterial Screening against Gram-positive bacteria (s); Figure S2: Antibacterial screening against Gram-negative bacteria (s); Figure S3: Antifungal Screening of thiazolidin-4-one derivatives of 1,3,4-oxadiazole/thiadiazole ring.

## Abbrevations

MIC	Minimum inhibitory concentration
MW	Molecular weight
IC_50_	Half-maximal inhibitory concentration
µM	Micromolar
M.pt.	Melting point
