# Supplementary figures and images for: 2-Substituted-3-(5-Substituted-1,3,4-oxadiazol/thiadiazol-2-yl) Thiazolidin-4-one Derivatives: Synthesis, Anticancer, Antimicrobial, and Antioxidant Potential
Source: Pharmaceuticals (Basel). 2023 May 29;16(6):805. doi: 10.3390/ph16060805 (PMC10305112; doi:10.3390/ph16060805)

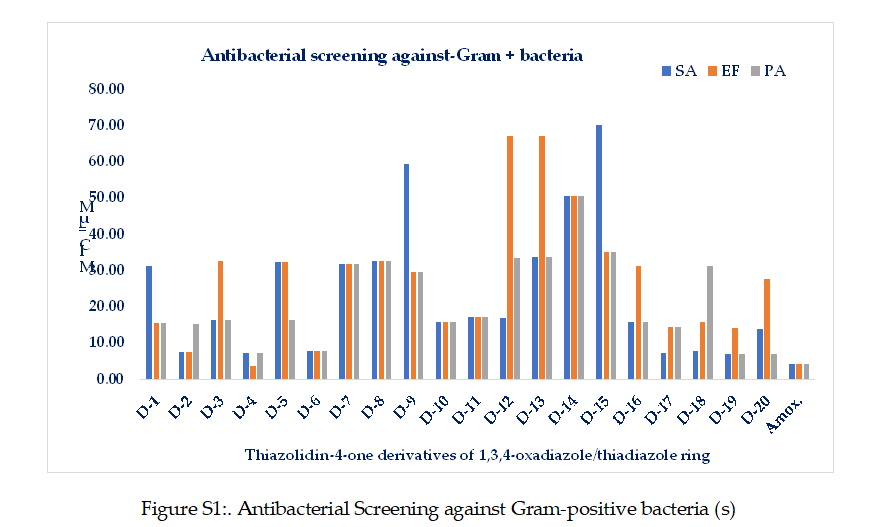

Supplement: Supplementary file 1 [file pharmaceuticals-16-00805-s001.zip › FIGURE S1.png]

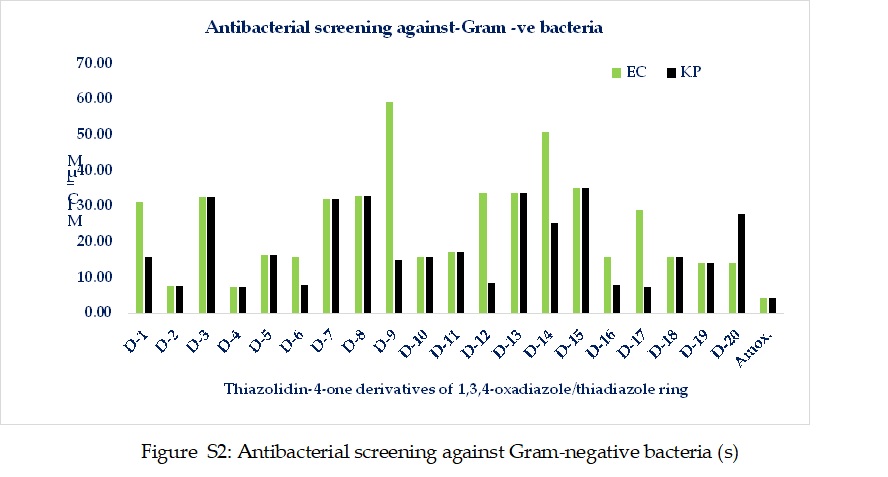

Supplement: Supplementary file 1 [file pharmaceuticals-16-00805-s001.zip › FIGURE S2.jpg]

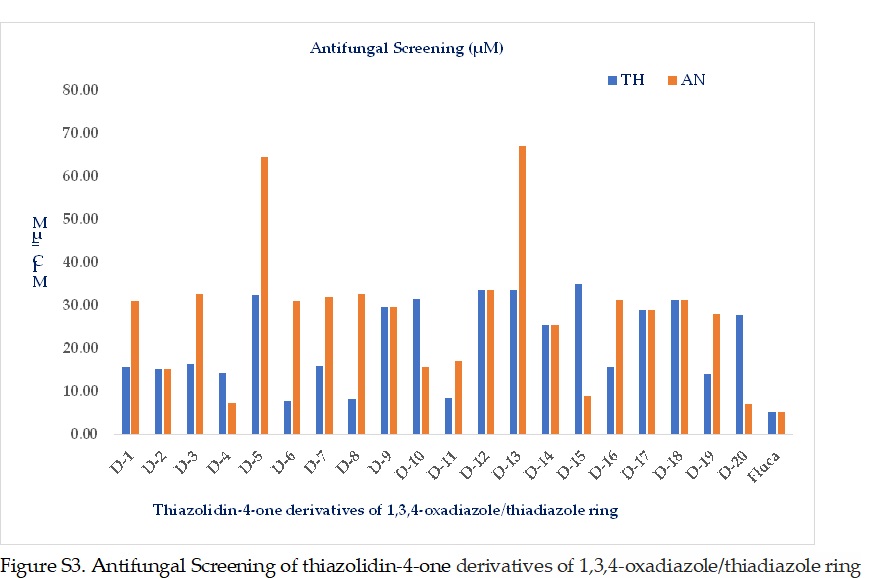

Supplement: Supplementary file 1 [file pharmaceuticals-16-00805-s001.zip › FIGURE S3.jpg]
